# Plasma taurine is an axonal excitability-translatable biomarker for amyotrophic lateral sclerosis

**DOI:** 10.1038/s41598-022-13397-6

**Published:** 2022-06-01

**Authors:** Tomoko Nakazato, Kazuaki Kanai, Tetsushi Kataura, Shuko Nojiri, Nobutaka Hattori, Shinji Saiki

**Affiliations:** 1 Department of Neurology, Juntendo University Graduate School of Medicine, 2-1-1 Hongo, Bunkyo-ku, Tokyo, 113-8421 Japan; 2 Department of Neurology, Fukushima Medical University, Fukushima, Japan; 3 Clinical Research Center, Juntendo University, Tokyo, Japan

**Keywords:** Metabolomics, Biomarkers, Motor neuron disease

## Abstract

Although various body fluid biomarkers for amyotrophic lateral sclerosis (ALS) have been reported, no biomarkers specifically reflecting abnormalities in axonal excitability indices have currently been established. Capillary electrophoresis time-of-flight mass spectrometry and liquid chromatography time-of-flight mass spectrometry were used to perform a comprehensive metabolome analysis of plasma from seven ALS patients and 20 controls, and correlation analysis with disease phenotypes was then performed in 22 other ALS patients. Additionally, electrophysiological studies of motor nerve axonal excitability were performed in all ALS patients. In the ALS and control groups, levels of various metabolites directly associated with skeletal muscle metabolism, such as those involved in fatty acid β-oxidation and the creatine pathway, were detected. Receiver operating characteristic curve analysis of the top four metabolites (ribose-5-phosphate, N6-acetyllysine, dyphylline, 3-methoxytyrosine) showed high diagnostic accuracy (area under the curve = 0.971) in the ALS group compared with the control group. Furthermore, hierarchical cluster analysis revealed that taurine levels were correlated with the strength-duration time constant, an axonal excitability indicator established to predict survival. No significant effects of diabetes mellitus and treatment (Riluzole and Edaravone) on this relationship were detected in the study. Therefore, plasma taurine is a potential novel axonal excitability-translatable biomarker for ALS.

## Introduction

Amyotrophic lateral sclerosis (ALS) is a partially hereditary, mainly sporadic, fatal neurodegenerative disease characterized by progressive muscular weakness of the cranial-bulbar region, trunk, and extremities caused by degeneration of both upper and lower motor neurons. Diagnostic criteria for ALS, such as the El Escorial and Awaji criteria, which are based on clinical characteristics as well as neurophysiological data, have been well-established^1,2^. To date, many epidemiological studies have shown factors related to the length of survival in ALS patients^3^. Specifically, older age and bulbar onset are reported to result in poorer outcomes. Psychosocial factors, dementia, nutritional status, respiratory function, and the speed of symptom progression are also related to outcome^3–6^. We previously reported that the indices of persistent sodium current and fast potassium conductance were significantly associated with survival in ALS patients, and moreover, that a longer strength-duration time constant (SDTC), which indicates an increased persistent sodium current in motor axons, was a strong predictor for ALS survival^7^. Although some metabolites in plasma have been suggested as potential diagnostic/surrogate biomarkers for ALS^8^, no plasma biomarkers closely linked to motor nerve axonal excitability indices specific for ALS have been reported.

Recent advances in metabolome analysis have enabled us and others to make important progress in understanding both the pathophysiology of various neurodegenerative diseases, such as Alzheimer’s disease and Parkinson’s disease, and how to more accurately diagnose them^9–13^. Additional advances also describe various metabolic changes in the blood of ALS patients^14–16^. Even with these advances, there are still no clinically established biomarkers that are supported by electrophysiological data such as motor nerve axonal excitability indices. In this context, we performed both comprehensive metabolome analyses and motor nerve axonal excitability testing in an attempt to combine these data.

## Results

### Metabolomics datasets

**Figure 1:**
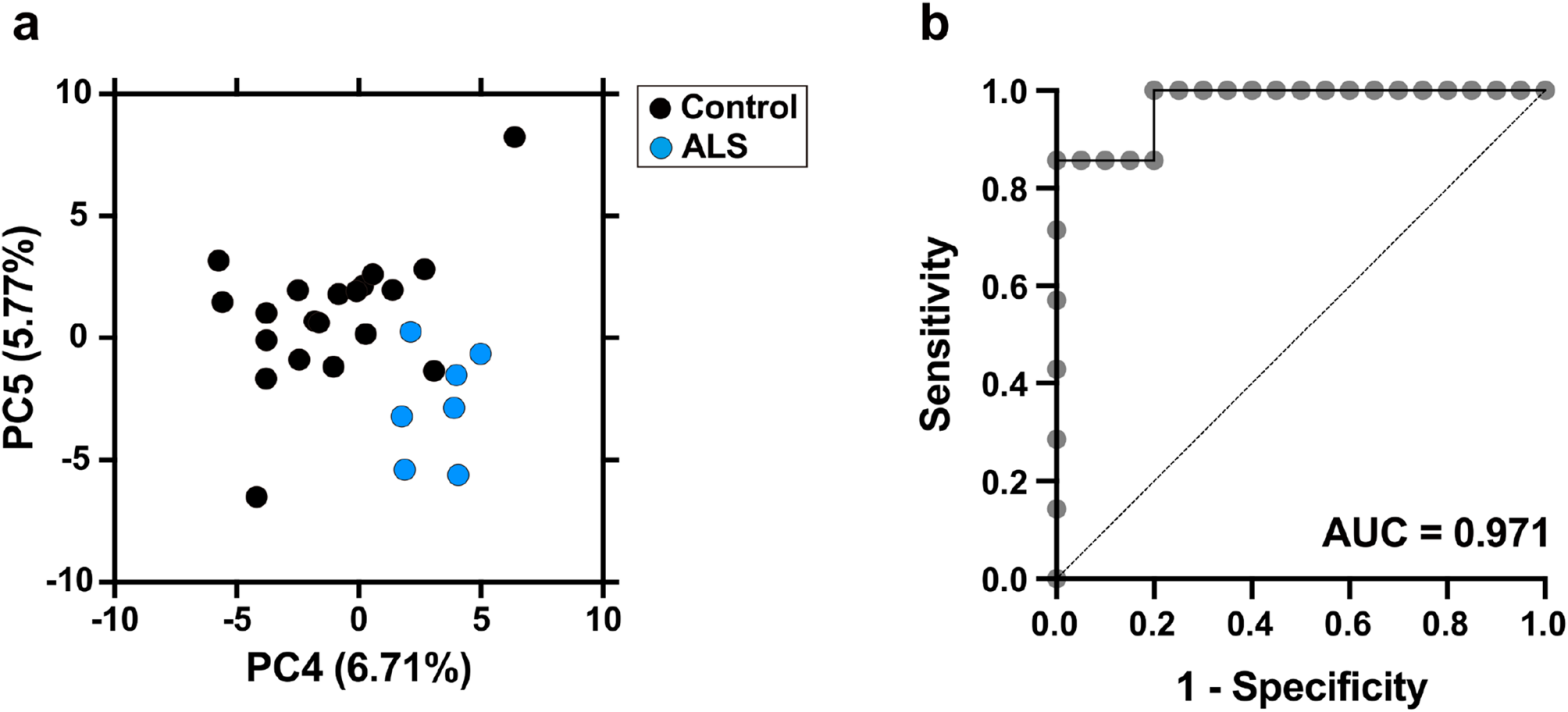
(**a**) Principal component analysis (PCA) of plasma metabolites in controls (n = 20) and amyotrophic lateral sclerosis (ALS) patients (first ALS group, n = 7). Two-dimensional plot of PCA scores of PC4 and PC5 in control subjects (black) versus ALS patients (blue) as analyzed according to the normalized values of plasma metabolites detected in participants in the pilot study. (**b**) Receiver operating characteristic (ROC) curve analysis using the values of the top four statistically significant metabolites (3-methoxytyrosine, dyphylline, N6-acetyllysine, and ribose-5-phosphate) from controls and ALS patients. *PC* principal component, *AUC* area under the curve.

The characteristics of 20 controls (Table 1) and seven ALS patients (first ALS group, Table 2) are shown. There were no significant differences in age between ALS patients and controls (*p* = 0.83). The body mass index (BMI) of ALS patients was less than that of controls, although this difference was not statistically significant (*p* = 0.48). On the basis of their *m/z* values, migration times, and retention time obtained with capillary electrophoresis time-of-flight mass spectrometry (CE-TOFMS) and liquid chromatography time-of-flight mass spectrometry (LC-TOFMS), 348 metabolites were detected in all controls and ALS patients. The 252 metabolites detected in > 50% of all participants were analyzed in detail. Principal component analysis (PCA) of the normalized metabolic data showed that the fourth principal component (PC4) and the fifth principal component (PC5) effectively discriminated ALS patients from controls (Fig. 1a). The top 30 positive and negative loading scores that were responsible for PC4 and PC5 are shown in Supplementary Table S1, S2, S3 and S4. Seventeen metabolites that were significantly changed in ALS patients compared with controls were identified (Table 3).

**Table 1:** Characteristics of control subjects (n = 20).

Number of controls	20
Male/female	11/9
Age, y, mean (SE)	61.8 (2.9)
Height, cm, mean (SE)	161.1 (2.5)
Body weight, kg, mean (SE)	59.3 (2.8)
BMI, mean (SE)	22.7 (0.7)
**Complications**	
Hypertension, n (%)	3 (15%)
Dyslipidemia, n (%)	6 (30%)
Hyperuricemia, n (%)	0
Other comorbidities	Headache 2 (7%), ET 1 (3%), RLS 1 (3%)

*SE* standard error, *BMI* body mass index, *ET* essential tremor, *RLS* restless legs syndrome.

**Table 2:** Characteristic of the first amyotrophic lateral sclerosis group (n = 7).

Number of patients	7
Male/female	2/5
Age, y, mean (SE)	63.0 (3.1)
Age at onset, y, mean (SE)	61.8 (3.1)
ΔFRS, mean (SE)	0.39 (0.07)
**Site of symptom onset**	
Bulbar	3
Upper limbs	3
Lower limbs	1
Height, cm, mean (SE)	162.3 (1,5)
Body weight, kg, mean (SE)	56.9 (3.9)
BMI, mean (SE)	21.6 (1.5)
%FVC, %, mean (SE)	92.2 (3.8)
CMAP amplitude in median nerve, mV, mean (SE)	5.5 (1.2)
**Nerve excitability properties**	
SDTC, ms, mean (SE)	0.46 (0.05)
TEd (10–30), %, mean (SE)	75.4 (2.0)
TEd (90–100), %, mean (SE)	57.1 (2.4)
TEh (90–100), %, mean (SE)	− 152.3 (5.8)
Supernormality, %, mean (SE)	− 33.7 (4.6)
**Complications**	
Hypertension, n (%)	0
Dyslipidemia, n (%)	0
Hyperuricemia, n (%)	0
Other comorbidities	Hashimoto disease 1 (10%)

*SE* standard error, *ΔFRS* progression rate (ratio of Functional Rating Scale score to time), *BMI* body mass index, *%FVC* percent forced vital capacity, *CMAP* compound *motor* action potential, *SDTC* strength duration time constant, *Ted* depolarising threshold electrotonus, *The* hyperpolarising threshold electrotonus.

**Table 3:** Statistically significant metabolites in seven amyotrophic lateral sclerosis (ALS) patients vs 20 controls.

Metabolites	ALS/control ratio	*p*-value
Ribose 5-phosphate	2.28	< 0.0001
N6-acetyllysine	0.35	< 0.0001
Dyphylline	1.36	< 0.0001
3-Methoxytyrosine	0.31	0.0001
7-Methylguanine	0.38	0.0003
Butyrylcarnitine	0.43	0.0013
N-Acetylgalactosamine/N-Acetylmannosamine/N-Acetylglucosamine	0.57	0.0016
N-Acetylputrescine	0.34	0.0039
Imidazole lactic acid	0.50	0.0040
Octanoylcarnitine	0.51	0.0061
Urocanic acid	0.47	0.0066
5-Oxoproline	0.83	0.0093
Creatinine	0.72	0.0093
Taurine	1.19	0.0112
Cystine	1.20	0.0263
Pelargonic acid	0.78	0.0478
Glutamine	1.11	0.0478

*P*-values were obtained by Wilcoxon’s test, comparison of ALS patients and controls.

### Skeletal muscle metabolism

#### Fatty acid β-oxidation

Fatty acid (FA) β-oxidation, which primarily occurs in skeletal muscle, produces long-chain acylcarnitines (LCACs). In detail, LCACs are formed from acyl-CoA by carnitine palmitoyltransferase 1 localized in the mitochondrial outer membrane and can either pass into the mitochondrial matrix via carnitine-acylcarnitine translocase or can exit the cell for disposal^17^. As shown in Table 3, acylcarnitines (butyrylcarnitine, octanoylcarnitine) and FAs (pelargonic acid, decanoic acid (ALS/control = 0.7 *p* = 0.0549), lauric acid (ALS/control = 0.88 *p* = 0.0716)) were observed at decreased levels in ALS patients, suggesting that FA β-oxidation as a whole is decreased.

#### Creatine metabolism

Creatine kinase catalyzes the reaction between creatine phosphate and adenosine 5′-diphosphate (ADP) to form creatine and adenosine 5-triphosphate (ATP). Creatinine is a breakdown product of creatine phosphate in muscle and is usually produced at a fairly constant rate by the body depending on muscle mass. Although creatine levels were not different between controls and ALS patients (*p* = 0.314), levels of creatinine were significantly suppressed in ALS patients when compared with controls (*p* = 0.0093) (Table 3), which is consistent with previous studies^18,19^. This implies that a high demand for skeletal muscle energy and reduced skeletal muscle volume are two pathophysiologies of ALS. The former would be more reasonable than the latter in ALS patients because no significant differences in methylhistidine (*p* = 0.263) and carnitine (*p* = 0.808) were found in this group. In addition, changes in creatine production would not contribute to the decrease of creatinine because there were no significant differences observed between ALS patients and controls in levels of arginine (*p* = 0.685), glycine (*p* = 0.263), or methionine (*p* = 0.533), which are indispensable for creatine production.

### Diagnostic values of the top four metabolites for ALS

In this study, we identified 17 metabolites with significantly different levels in ALS patients compared with controls (Table 3). Therefore, we next examined the diagnostic power of the top four metabolites (ribose-5-phosphate, N6-acetyllysine, dyphylline, and 3-methoxytyrosine) in 20 controls and seven ALS patients. We performed multiple regression analyses to evaluate the diagnostic value of the metabolite set and described the appropriate receiver operating characteristic curves with area under the curve (AUC) values. The set including the four metabolites could differentiate ALS samples from control samples with high efficiency (AUC = 0.971, sensitivity = 0.857, specificity = 1) (Fig. 1b). These data indicate that ALS patients can be differentiated from controls by this set of plasma metabolites.

### Association of clinical parameters with metabolites

We retrospectively analyzed 66 ALS patients to assess survival (Supplementary Table S5). The univariate Cox regression analysis results are shown in Supplementary Table S6. There was no significant difference in the percentage of patients who received each treatment in the subgroups categorized by excitability index. Consistent with a previous report^7^, the SDTC was significantly correlated with survival in ALS patients (*p* = 0.0471) (Supplementary Table S6, Supplementary Fig. S1). When four patients with diabetic polyneuropathy (moderate-severe) and three patients treated with Riluzole and/or Edaravone were excluded from the nerve excitability analysis, the SDTC remained a strong predictor for survival (hazard ratio, HR, 2.31; 95% confidence interval, CI, 1.02 to 5.23; *p* = 0.0446). A prolonged SDTC was strongly associated with shorter survival time.

**Figure 2:**
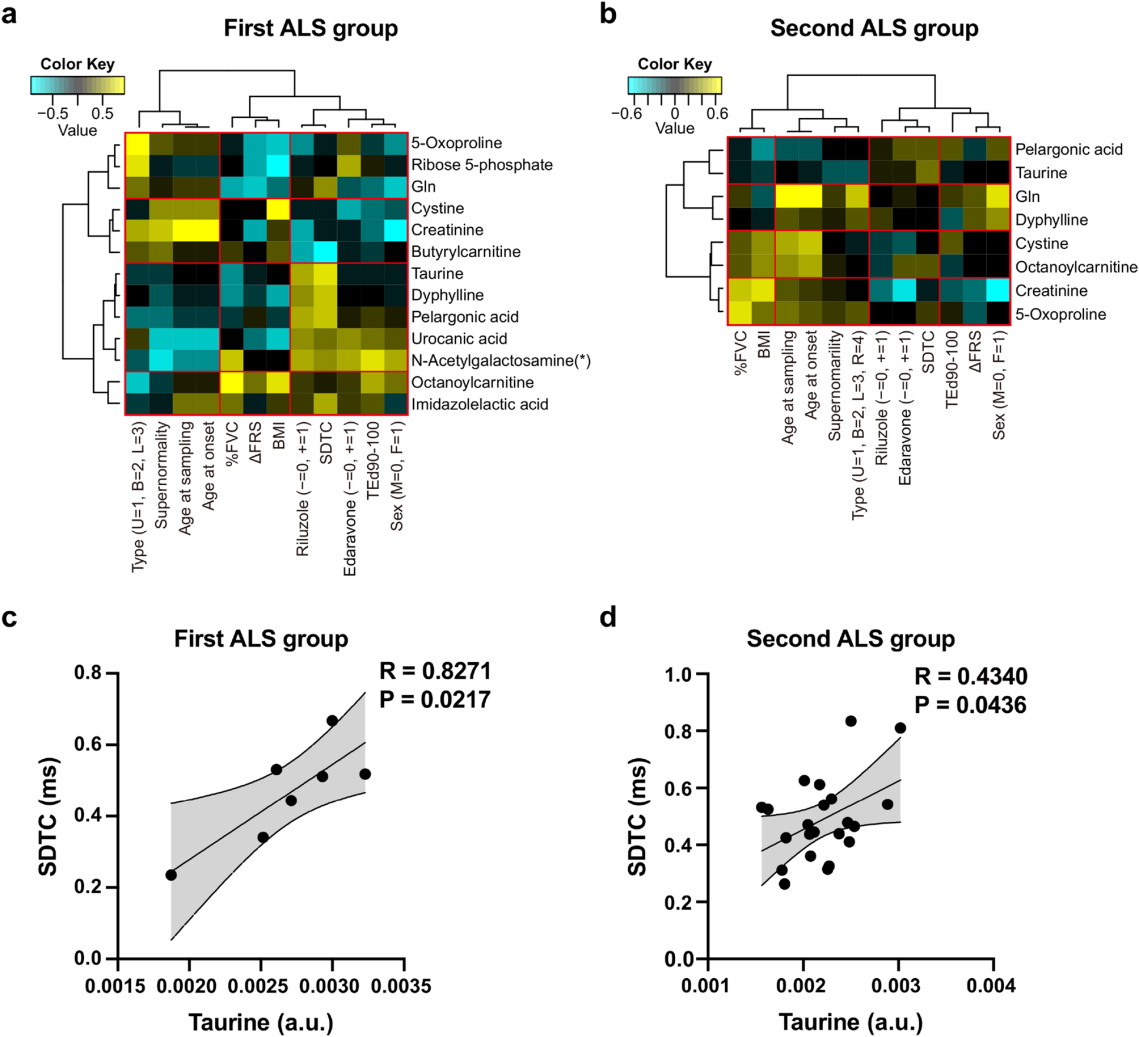
(**a**,**b**) Hierarchical clustering analysis with heatmap representation of the metabolites and the clinical parameters of (**a**) the first amyotrophic lateral sclerosis (ALS) group and (**b**) the second ALS group. Rows indicate (**a**) 13 metabolites with statistical significance between the control and first ALS group and (**b**) a subset of the 13 metabolites detected in the second ALS group. The N-acetylgalactosamine(*) row includes N-acetylmannosamine and N-acetylglucosamine. 3-Methoxytyrosine, 7-methylguanine, N-acetylputrescine, and N6-acetyllysine were excluded from the dataset because the four metabolites had a distribution along a vertical line, and thus no correlation coefficient was available. Columns indicate the 12 clinical parameters assessed in the study. (**a**,**b**) The heatmap shows a gradient color scale ranging from cyan to black to yellow, indicating the normalized score (Z-score) calculated from Spearman’s rank correlation coefficient for each combination. (**c**,**d**) Correlation analysis between taurine and nerve excitability properties (SDTC) in both groups of ALS patients. Scatterplots show the correlation between taurine and SDTC in the (**c**) first ALS group (n = 7) and (**d**) second ALS group (n = 22). Pearson’s correlation coefficient (R) and the p-value (*p*) are shown. *a.u.* arbitrary unit, *BMI* body mass index, *FA* fatty acid, *%FVC* percent forced vital capacity, *SDTC* strength duration time constant; sex (*M* male, *F* female), *T2DM* type 2 diabetes mellitus, *Ted* depolarizing threshold electrotonus; treatment with Riluzole and/or Edaravone (− negative/non-administration, +  positive/ administration); type = site of symptom onset (*U* upper limbs, *B* bulbar, *L* lower limbs, *R* respiratory failure), *ΔFRS* progression rate (ratio of Functional Rating Scale score to time).

Next, to identify ALS-specific metabolites reflecting clinical variables and axonal excitability indices, we performed hierarchical clustering analysis (HCA) on clinical variables previously suggested as biomarkers for disease severity and 13 metabolites that were significantly different between controls and ALS patients in seven patients (first ALS group). In ALS patients, the HCA with heat map analysis showed that taurine, pelargonic acid, and three other metabolites were positively correlated with the SDTC, a predictor for survival (Fig. 2a). Other clinical variables such as sex, drug administration (Edaravone, Riluzole), and the depolarizing threshold electrotonus (TEd)90–100, showed similar correlation patterns to the SDTC (Fig. 2). As described above, we confirmed that the SDTC was a predictor for survival. Therefore, we speculate that some metabolites related to the SDTC can be useful blood biomarkers. To validate these observations, we further recruited 22 ALS patients (second ALS group) (Table 4) and performed metabolomic and the same statistical analyses. HCA with heatmap analysis on eight metabolites (a subset of the 17 metabolites detected in the second ALS group) identified two metabolites (taurine and pelargonic acid) that were positively correlated with the SDTC (Fig. 2b). Likewise, drug administration (Edaravone, Riluzole) was also grouped in the same cluster with the SDTC (Fig. 2b). Taurine and pelargonic acid displayed positive correlations in both cohorts, with taurine having greater statistical significance (Fig. 2b). A positive correlation of taurine with the SDTC was clearly identified in both ALS groups (Fig. 2c,d).

**Table 4:** Characteristic of the second amyotrophic lateral sclerosis group (n = 22).

Number of patients	22
Male/Female	10/12
Age, y, mean (SE)	62.7 (3.1)
Age at onset, y, mean (SE)	61.0 (3.2)
ΔFRS, mean (SE)	0.57 (0.09)
**Site of symptom onset**	
Bulbar	4
Upper limbs	11
Lower limbs	6
Respiratory	1
Height, cm, mean (SE)	161.1 (1.9)
Body weight, kg, mean (SE)	51.8 (2.1)
BMI, mean (SE)	19.9 (0.7)
%FVC, %, mean (SE)	87.7 (5.6)
CMAP amplitude in median nerve, mV, mean (SE)	5.8 (0.6)
**Nerve excitability properties**	
SDTC, ms, mean (SE)	0.49 (0.03)
TEd (10–30), %, mean (SE)	75.6 (1.4)
TEd (90–100), %, mean (SE)	54.1 (1.7)
TEh (90–100), %, mean (SE)	− 136.9 (6.4)
Supernormality, %, mean (SE)	− 30.4 (2.0)
**Complications**	
Hypertension, n (%)	8 (36%)
Dyslipidemia, n (%)	5 (22%)
Hyperuricemia, n (%)	1 (3%)
Other comorbidities	PBC 1 (5%)

*SE* standard error, *ΔFRS* progression rate (ratio of Functional Rating Scale score to time), *BMI* body mass index, *%FVC* percent forced vital capacity, *CMAP* compound *motor* action potential, *PBC* primary biliary cholangitis, *SDTC* strength duration time constant, *Ted* depolarising threshold electrotonus, *The* hyperpolarising threshold electrotonus.

### Effects of type 2 diabetes mellitus (T2DM) on biomarker selection

Diabetic neuropathy is characterized by various forms of peripheral nerve damage such as mononeuropathy, cranial neuropathy, autonomic neuropathy, and distal sensory neuropathy with complexed phenotypes including demyelination and axonal dysfunction^20^. Because significant changes in the SDTC have been detected in T2DM patients with polyneuropathy^21–24^, we re-analyzed the data from 25 ALS patients comprising the second ALS group (n = 22) and ALS patients complicated with T2DM (n = 3, recruited concurrently with the second ALS group). PCA of plasma metabolites showed that no principal component effectively discriminated patients without T2DM from patients with T2DM (Supplementary Fig. S2a). In addition, no significant difference in eight metabolites, which comprised a subset of the 17 metabolites determined to be significantly different between the control group and the first ALS group, was found in the second group, and no significant difference in the SDTC was found between patients with and without T2DM (Supplementary Fig. S2b, c). Furthermore, Heatmap analysis and correlation analysis of the 25 ALS patients showed a significant positive correlation between taurine and the SDTC (*p* = 0.0255) (Supplementary Fig. S2d, e). Taken together, the results suggested that T2DM did not affect biomarker selection.

### Effects of Riluzole and Edaravone on biomarker selection

When blood samples were obtained, four of seven patients (first ALS group) and six of 22 patients (second ALS group) were being treated with Edaravone and/or Riluzole. To examine the effects of Edaravone or Riluzole treatment, we performed a validation study using the second ALS group. The second ALS group was divided into ALS patients who received Edaravone and/or Riluzole treatment (n = 6) and ALS patients who were not treated with Edaravone or Riluzole (n = 16). First, PCA of the normalized metabolic data showed that no principal component effectively discriminated between patients with and without Edaravone/Riluzole treatment (Supplementary Figure S3a). Next, the eight metabolites described above and nerve excitability properties (SDTC) were shown to be not significantly different between patients with and without Edaravone/Riluzole treatment (Supplementary Fig. 3Sb, c). The results suggested that the drugs had no effect on biomarker selection.

## Discussion

In the current study, the levels of 17 metabolites primarily from skeletal muscle metabolism, such as FA β-oxidation and creatine metabolism, were significantly altered in the serum of ALS patients compared with controls. Using the top four metabolites with the highest statistical significance, ALS patients were sufficiently differentiated from controls. In particular, we identified taurine as a survival-associated, axonal excitability-translatable biomarker of ALS. The taurine levels in both ALS groups were correlated with the SDTC, which predicts survival, as examined in 66 ALS patients. Taken together, the amount of taurine in plasma has great potential as a non-invasive, axonal degeneration-dependent marker for ALS.

Creatine is mainly catabolized from several essential amino acids such as glycine, arginine, and methionine, in skeletal and cardiac muscle^25^. Creatinine formed from creatine phosphate can pass through the cell membrane and finally into the blood. Recently, a 49% increase of plasma creatine, with associated 20% and 24% decreases in creatinine and methylhistidine, respectively, was reported in the serum of ALS patients^16^, consistent with previous reports^18,19^. Additionally, serum creatinine levels were related to clinical progression and a worse time-to-death prognosis^18,19,26^. Furthermore, the serum creatinine levels of ALS patients were significantly suppressed^18,19,27,28^, which is consistent with the findings of our study. Moreover, ALS is associated with hypermetabolism, which is linked to defects in muscle mitochondrial energy metabolism, such as ATP depletion and increased oxygen consumption^29,30^. Taken together, skeletal muscles in ALS patients produce high energy levels (ATP) by conversion of creatine phosphate to creatine; however, with the reduction of the substrate, creatine phosphate, the production of creatinine is decreased.

FA β-oxidation mostly occurs in the skeletal muscles. In this study, several FAs and acylcarnitines were decreased in ALS patients compared with controls. We expected that these changes were associated with denervation-linked muscle wasting. However, factors other than atrophy caused by denervation contribute to these changes because methylhistidine and carnitine are related to muscle volume and were not significantly changed^31^. ALS is associated with several energy metabolism defects, including weight loss, hypermetabolism, and hyperlipidemia^29^. Collectively, the decreased FA β-oxidation in this study was presumed to be caused by several factors including muscle wasting.

In this study, plasma levels of taurine in ALS patients were significantly correlated with the SDTC, which is reported as a measure of persistent sodium current^7,32^. The clinical significance of the SDTC in T2DM patients is still controversial; however, the relationship of taurine with the SDTC was preserved, even in ALS patients with T2DM. Additionally, an effect of treatment (Edaravone and/or Riluzole) on the metabolic profile was not detected. In experimental ALS models, several neuroprotective effects of taurine on glutamate cytotoxicity, oxidative stress, and sodium channel dysfunction have been suggested^33–35^. In addition, in postmortem analyses, taurine was significantly increased in the cortex and spinal cord of ALS patients compared with controls^36,37^. Several reports have suggested a relationship between taurine and axons. In the hippocampus, taurine could provoke an increase in axon excitability^38^. In an experimental model, taurine could promote axon regeneration after spinal cord injury^39^. Moreover, nerve injury might lead to changes in axolemmal sodium channel distribution that could account for neuropathic hyperexcitability. Previous excitability studies have suggested two types of axonal abnormalities in ALS: increased persistent sodium currents and reduced potassium currents in motor axons, both of which lead to axonal hyperexcitability^40–42^. Consequently, taurine might be directly or indirectly related to axon hyperexcitability. Furthermore, taurursodiol, which is taurine-conjugated to ursodeoxycholic acid, has been shown to attenuate neuronal death^43^. Hence, a recent trial of sodium phenylbutyrate–taurursodiol indicated that it resulted in a slower functional decline in ALS patients^44^. Regardless of whether plasma taurine can pass through the blood-nerve or blood–brain barriers and directly stimulate neuronal membranes and/or transporters, compensatory upregulation of plasma taurine might occur to lessen axonal damage in ALS patients.

Our study has several limitations. Diagnoses of ALS were acquired in a single university hospital. Likewise, blood sampling was performed at various clinical disease stages of ALS. In the power analysis^45^, the power of each metabolite was less than 0.8 (Supplementary Table S7), which indicated that our sample size was low. Therefore, our study is characterized as a pilot study because of the small sample size. In future studies, more than 100 patients should be assessed to ascertain results. While we did not perform excitability testing in control subjects in this study because previous reports noted that the SDTC was higher in ALS patients than that in controls^40,46,47^, the correlation between taurine and the SDTC in control subjects should be determined to show that taurine is an ALS-specific survival marker. Survival analysis using taurine is currently under investigation. Further studies should examine the exact molecular mechanisms of the association between taurine elevation and axonal sodium channel abnormalities.

## Methods

### Ethics statement

This study protocol complied with the Declaration of Helsinki and was approved by the ethics committee of Juntendo University (#2012157). Written informed consent has been given by all participants.

### Participants

Sixty-six patients with ALS, who were diagnosed according to the revised El Escorial criteria^1^ and/or Awaji criteria^2^ for definite or probable ALS at Juntendo University Hospital from 2013–2018, were included in this study. No patients had a history of peripheral neuropathy, spondylosis with a cervical spinal surgery, or collagen vascular diseases. Additionally, ALS patients with unrecordable median motor responses or with carpal tunnel syndrome were excluded. Controls without skeletal muscle disease and a history of malignant tumor, aspiration pneumonia or inflammatory diseases including collagen vascular diseases were included. The pilot study consisted of 30 age-matched controls and 10 ALS patients. Thirty ALS patients were included in the validation study to confirm the significance of biomarker candidates. Patients with cardiovascular diseases, stroke, dementia, or diabetes have altered metabolomic characteristics^48–51^. Therefore, ALS patients with hematological diseases, diabetes, lung disorders, heart diseases, previous stroke, or ALS related-dementia were excluded from the metabolomics analysis. Controls with hematological diseases, diabetes, lung disorders, heart diseases, or previous stroke were also excluded. Hence, metabolomic and further analyses were performed on 20 controls and seven ALS patients (first ALS group) in the pilot study and 22 ALS patients (second ALS group) in the validation study. In addition, we retrospectively analyzed 66 ALS patients to assess survival (Supplementary Table S5).

### Sample collection

Sample collection for metabolomics analysis was performed from December 2014 to January 2017. Plasma was extracted as described previously^11^. Sample preparation and immediate mass spectrometry analysis in the pilot study and validation study were performed in February 2015 and February 2019, respectively.

### Metabolite extraction

Metabolite extraction and metabolome analyses for both studies were conducted at Human Metabolome Technologies (HMT: Tsuruoka Yamagata, Japan). The detailed method used for the pilot study (control and first ALS group) was described in a previous report^11^. The following method was used for the validation study (second ALS group). Briefly, 50 µL of plasma was added to 450 µL of methanol containing internal standards (Solution ID: H3304-1002, Human Metabolome Technologies, Inc., Tsuruoka, Japan) at 0 °C to inactivate enzymes. The extract solution was thoroughly mixed with 500 µL of chloroform and 200 µL of Milli-Q water and centrifuged at 2,300 × *g* at 4 °C for 5 min. Then, 350 µL of the upper aqueous layer was centrifugally filtered through a Millipore 5-kDa cut-off filter to remove proteins. The filtrate was centrifugally concentrated and resuspended in 50 µL of Milli-Q water for metabolome analysis at HMT.

### Metabolome analysis

Metabolome analysis was conducted with the *Basic Scan* package of HMT using CE-TOFMS based on methods described previously^52,53^. Briefly, CE-TOFMS analysis was carried out using an Agilent CE capillary electrophoresis system equipped with an Agilent 6210 time-of-flight mass spectrometer, Agilent 1100 isocratic HPLC pump, Agilent G1603A CE-MS adapter kit, and Agilent G1607A CE-ESI–MS sprayer kit (Agilent Technologies, Santa Clara, CA, USA). The systems were controlled by Agilent G2201AA ChemStation software version B.03.01 for CE (Agilent Technologies) and connected by a fused silica capillary (50 µm *i.d.* × 80 cm total length) with commercial electrophoresis buffer (H3301-1001 and H3302-1023 for cation and anion analyses, respectively, HMT) as the electrolyte. The samples were scanned from *m/z* 50 to 1000^52^. Peaks were extracted using MasterHands automatic integration software (Keio University, Tsuruoka, Yamagata, Japan) to obtain peak information including the *m/z*, peak area, and migration time (MT)^54^. Signal peaks corresponding to isotopomers, adduct ions, and other product ions of known metabolites were excluded, and the remaining peaks were annotated according to the HMT metabolite database based on their *m*/*z* values and MTs. The areas of the annotated peaks were then normalized based on internal standard levels and sample volumes to obtain the relative levels of each metabolite. Detected metabolites were plotted on metabolic pathway maps using VANTED software^55^.

### Nerve excitability testing

Multiple excitability measurements were performed on the median nerve at the wrist using a computerized program (QTRAC with multiple excitability protocols: TRONDNF and TRONDXM2, Institute of Neurology, London, UK)^32,56^. The compound muscle action potential was recorded from the abductor pollicis brevis after median nerve stimulation at the wrist. Excitability testing was conducted after sufficient skin was scraped to reduce skin impedance. During testing, the skin temperature near the stimulus site was maintained at > 32 °C. The following excitability indices were included: SDTC (XM2-protocol), threshold electrotonus, refractoriness, supernormality, and late subnormality of the recovery cycle (TRONDNF). We chose the values of SDTC and supernormality in the recovery cycle as electrophysiological prognostic factors.

### Statistical analysis

Data analysis of metabolites was performed according to the following approach. When a value was under the limit of detection, we assigned it half the minimum value of its compound. First, we used Wilcoxon tests to compare the differences between the ALS (first ALS group, n = 7) and control groups (n = 20). The ratio of each compound in ALS patients and controls was examined, and *p* < 0.05 was considered statistically significant. Second, Spearman’s rank correlation coefficient was used to assess the correlations among clinical data, nerve excitability properties, and significantly altered metabolites in the first ALS group. To investigate consequences of the observed correlations, we visualized them in a heat map representation and performed hierarchical clustering analysis using the correlation distance and Ward’s method. The correlation was also examined in the second ALS group (n = 22) using Spearman’s rank correlation coefficient and a heat map representation. Finally, the correlation between taurine and the SDTC was further examined using Pearson’s correlation coefficient. Additionally, survival analysis was performed using clinical data and nerve excitability properties. Overall survival was defined as the time from symptom onset until death, tracheostomy, or noninvasive positive pressure ventilation for more than 23 h a day. For time-to-event outcomes, the time to first event durations were compared using the log-rank test, while the Kaplan–Meier method was used to estimate the absolute risk of each event for each group. HRs and 95% CIs were estimated using the Cox proportional hazard model. For excitability indices, ALS patients were divided into two subgroups with higher or lower values according to cut-off values (SDTC: 0.41 ms, supernormality: − 23.4%), the mean values of the control subjects, as previously reported^7^. After diagnosis, 65 patients were treated with Riluzole, 49 patients were treated with Edaravone, and 10 patients were treated with mecobalamin (50 mg, intramuscular). We used R (version 4.1.2) for PCA and HCA, and JMP16 (SAS Institute, Tokyo, Japan) statistical software was used for all other data analyses.

## Supplementary Information

Supplementary Information.

## Data Availability

The data produced from this study, including the clinical characteristics, metabonomic data, and measurements of nerve excitability, can be requested in Microsoft Excel format from the corresponding author.
