# Supplementary material for: Plasma taurine is an axonal excitability-translatable biomarker for amyotrophic lateral sclerosis
Source: Sci Rep. 2022 Jun 1;12:9155. doi: 10.1038/s41598-022-13397-6 (PMC9160240; doi:10.1038/s41598-022-13397-6)
Supplement: Supplementary file 1 — Supplementary Information. [file 41598_2022_13397_MOESM1_ESM.pdf]

Supplementary Tables and Figures

# Plasma taurine is an axonal excitability-translatable biomarker for amyotrophic lateral sclerosis

Tomoko Nakazato[1], Kazuaki Kanai[2], Tetsushi Kataura[1], Shuko Nojiri[3], Nobutaka Hattori[1], Shinji Saiki[1]*

Affiliations:

1. Department of Neurology, Juntendo University Graduate School of Medicine

2. Department of Neurology, Fukushima Medical University

3. Clinical Research Center, Juntendo University

*Correspondence to: Dr. Shinji Saiki. Department of Neurology, Juntendo University Graduate School of Medicine, 2-1-1 Hongo, Bunkyo-ku, Tokyo 113-8421, Japan; TEL: 81-3-3813-3111; FAX: 81-3-5800-0547; E-mail: ssaiki@juntendo.ac.jp

Supplementary Table S1. Metabolites and positive loading scores in PC4.

| Metabolites | Loading scores |
|---|---|
| Hypotaurine | 0.162 |
| Ribose 5-phosphate | 0.158 |
| N-Acetylglycine | 0.156 |
| Serine | 0.153 |
| Acylcarnitine (18:1) | 0.144 |
| Palmitoylcarnitine | 0.142 |
| Dyphylline | 0.130 |
| Aspartic Acid | 0.126 |
| 1-Methyladenosine | 0.124 |
| N2-Phenylacetylglutamine | 0.123 |
| Acylcarnitine (18:2)-1 | 0.119 |
| 2-Hydroxybutyric acid | 0.119 |
| Glycine | 0.107 |
| 3-Indoxylsulfuric acid | 0.102 |
| Carnitine | 0.100 |
| Glutamine | 0.098 |
| Phenylalanine | 0.095 |
| Methionine | 0.091 |
| Leucine | 0.090 |
| Cysteine glutathione disulfide | 0.089 |
| Theobromine | 0.088 |
| Acylcarnitine (16:1) | 0.088 |
| Glycochenodeoxycholic acid | 0.086 |
| Proline | 0.084 |
| Hypoxanthine | 0.080 |
| Ethanolamine | 0.078 |
| Asparagine | 0.076 |
| 18-Hydroxycorticosterone Cortisol | 0.075 |
| Phenylalanine- Glutamine | 0.073 |
| Heptadecanoic acid | 0.070 |

Abbreviations: PC = principal component

Supplementary Table S2. Metabolites and negative loading scores in PC4.

| Metabolites | Loading scores |
|---|---|
| Mucic acid | -0.208 |
| Pipecolic acid | -0.200 |
| 5-Oxoproline | -0.188 |
| Imidazolelactic acid | -0.185 |
| Thiaproline | -0.176 |
| Decanoic acid | -0.172 |
| Pelargonic acid | -0.162 |
| Fatty acid (14:3) | -0.150 |
| Etiocholan-3α-ol-17-one sulfate-2 | -0.139 |
| Glycerol | -0.129 |
| 7-Dehydrocholesterol | -0.114 |
| 3-Methoxytyrosine | -0.113 |
| Betaine | -0.111 |
| Etiocholan-3α-ol-17-one sulfate-1 | -0.106 |
| Stachydrine | -0.105 |
| Deoxycholic acid | -0.104 |
| Palmitoylethanolamide | -0.104 |
| Oleoyl ethanolamine | -0.100 |
| Methionine sulfoxide | -0.096 |
| Cholesterol sulfate | -0.092 |
| N-Methylproline | -0.092 |
| N-Acetylputrescine | -0.082 |
| Caffeine | -0.081 |
| Lactic acid | -0.079 |
| Isethionic acid | -0.075 |
| 2-Hydroxyvaleric acid | -0.074 |
| Phosphorylcholine | -0.074 |
| Glyceric acid | -0.073 |
| Lauric acid | -0.073 |
| cis-4,7,10,13,16,19-Docosahexaenoic acid | -0.071 |

Abbreviations: PC = principal component

Supplementary Table S3. Metabolites and positive loading scores in PC5.

| Metabolites | Loading scores |
|---|---|
| Cysteine glutathione disulfide | 0.185 |
| N-Acetylglycine | 0.180 |
| Serine | 0.166 |
| Isoleucine-Alanine | 0.162 |
| Urocanic acid | 0.158 |
| Glycine | 0.151 |
| Butyrylcarnitine | 0.148 |
| Proline | 0.143 |
| N-Acetylgalactosamine/N-Acetylmannosamine/ N-Acetylglucosamine | 0.142 |
| 5-Hydroxylysine | 0.141 |
| 7-Methylguanine | 0.139 |
| 3-Methoxytyrosine | 0.135 |
| Triethanolamine | 0.130 |
| Lauric acid | 0.125 |
| Malic acid | 0.113 |
| Aspartic Acid | 0.104 |
| 3-Aminobutyric acid | 0.103 |
| Isobutyrylcarnitine | 0.101 |
| ADP | 0.097 |
| ADMA | 0.080 |
| N6-Acetyllysine | 0.073 |
| N-Acetylalanine | 0.069 |
| Quinic acid | 0.068 |
| S-Methylcysteine | 0.067 |
| SDMA | 0.065 |
| Glutamic Acid | 0.064 |
| Fatty acid (14:3) | 0.063 |
| Pyruvic acid | 0.061 |
| Acylcarnitine (18:2)-1 | 0.060 |
| Guanidoacetic acid | 0.058 |

Abbreviations: PC = principal component

Supplementary Table S4. Metabolites and negative loading scores in PC5.

| Metabolites | Loading scores |
|---|---|
| Taurine | -0.221 |
| Urea | -0.198 |
| Dyphylline | -0.183 |
| cis-4,7,10,13,16,19-Docosahexaenoic acid | -0.147 |
| Isethionic acid | -0.141 |
| Ethanolamine | -0.126 |
| Cystine | -0.126 |
| Stachydrine | -0.125 |
| Ribose 5-phosphate | -0.122 |
| cis-5,8,11,14,17-Eicosapentaenoic acid<br>Fatty acid(20:5) | -0.117 |
| Guanidinosuccinic acid | -0.113 |
| Glycerol | -0.112 |
| Indole-3-acetic acid | -0.110 |
| Paraxanthine | -0.110 |
| Uridine | -0.110 |
| Ascorbate 2-sulfate | -0.109 |
| Phenylalanine | -0.105 |
| Tryptophan | -0.103 |
| Caffeine | -0.097 |
| N-Methylproline | -0.094 |
| 1-Methylhistidine 3-Methylhistidine | -0.089 |
| Myristoleic acid | -0.088 |
| α-Tocopherol | -0.088 |
| Tyrosine | -0.087 |
| Lysine | -0.086 |
| Decanoic acid | -0.086 |
| 18-Hydroxycorticosterone Cortisol | -0.083 |
| Choline | -0.083 |
| Glutamine | -0.081 |
| Fatty acid (22:5) | -0.080 |

Abbreviations: PC = principal component

Supplementary Table S5. Characteristic of amyotrophic lateral sclerosis patients (n = 66)

| | |
|---|---|
| Number of patients with ALS | 66 |
| Male/Female | 37/29 |
| Age at onset, y, mean (SE) | 60.0 (1.6) |
| ΔFRS, mean (SE) | 0.59 (0.06) |
| Site of symptom onset | |
| Bulbar | 15 |
| Upper limbs | 32 |
| Lower limbs | 17 |
| Respiratory | 1 |
| Combined | 1 |
| Hight, cm, mean (SE) | 162.9 (1.4) |
| Body weight, kg, mean (SE) | 56.5 (1.6) |
| BMI, mean (SE) | 21.1 (0.45) |
| %FVC, %, mean (SE) | 83.2 (3.1) |
| CMAP amplitude in median nerve, mV, mean (SE) | 5.8 (0.4) |
| Nerve Excitability properties | |
| SDTC, ms, mean (SE) | 0.49 (0.02) |
| Supernomality, %, mean (SE) | −30.7 (1.4) |
| Complications | 23 (34%) |
| Hypertension, n (%) | 14 (21%) |
| Diabetes , n (%) | 11 (17%) |
| Dyslipidemia, n (%) | 1 (2%) |
| Hyperuricemia, n (%) | 1 (2%) |
| Other comorbidities | Dementia 6 (10%), Heart disease 4(7%), Hashimoto disease 1 (2%), PBC 1 (2%) |

Abbreviations: SE = standard error; ΔFRS = progression rate (ratio of Functional Rating Scale score to time); BMI = body mass index; % FVC = percent forced vital capacity; CMAP = compound motor action potential; PBC = primary biliary cholangitis; SDTC = strength duration time constant

Supplementary Table S6. Univariate analysis of prognostic factor in ALS patients (n = 66)

| Category | | HR | 95% CI Lower bound | 95% CI Upper bound | *p* |
|---|---|---|---|---|---|
| Age at onset | > 60 y.o. | 1.89 | 0.99 | 3.62 | 0.0525 |
| ΔFRS | > 0.458 | 2.63 | 1.38 | 4.93 | 0.0031 |
| %FVC | < 80 % | 2.69 | 1.40 | 5.16 | 0.0029 |
| CMAP | < 5 mV | 1.83 | 1.01 | 3.30 | 0.0451 |
| SDTC | > 0.41 ms | 2.26 | 1.01 | 5.08 | 0.0471 |
| Supernomality | > −23.4 % | 1.35 | 0.64 | 2.85 | 0.4302 |

Abbreviations: HR = hazard ratio; CI = confidence interval; ΔFRS = progression rate (ratio of Functional Rating Scale score to time); % FVC = percent forced vital capacity; CMAP = compound motor action potential; SDTC = strength duration time constant

Supplementary Table S7.

Statistically significant metabolites and power analysis in seven amyotrophic lateral sclerosis (ALS) patients vs 20 controls

| Metabolites | ALS/control ratio | *p*-value | Power |
|---|---|---|---|
| Ribose 5-phosphate | 2.28 | < 0.0001 | 0.014 |
| N6-Acetyllysine | 0.35 | < 0.0001 | 0.012 |
| Dyphylline | 1.36 | < 0.0001 | 0.015 |
| 3-Methoxytyrosine | 0.31 | 0.0001 | 0.009 |
| 7-Methylguanine | 0.38 | 0.0003 | 0.015 |
| Butyrylcarnitine | 0.43 | 0.0013 | 0.040 |
| N-Acetylgalactosamine/ N-Acetylmannosamine/ N-Acetylglucosamine | 0.57 | 0.0016 | 0.051 |
| N-Acetylputrescine | 0.34 | 0.0039 | 0.063 |
| Imidazolelactic acid | 0.50 | 0.0040 | 0.066 |
| Octanoylcarnitine | 0.51 | 0.0061 | 0.056 |
| Urocanic acid | 0.47 | 0.0066 | 0.077 |
| 5-Oxoproline | 0.83 | 0.0093 | 0.101 |
| Creatinine | 0.72 | 0.0093 | 0.097 |
| Taurine | 1.19 | 0.0112 | 0.046 |
| Cystine | 1.20 | 0.0263 | 0.145 |
| Pelargonic acid | 0.78 | 0.0478 | 0.222 |
| Glutamine | 1.11 | 0.0478 | 0.181 |

*P*-values were obtained by Wilcoxon's test, comparison of ALS patients and controls.

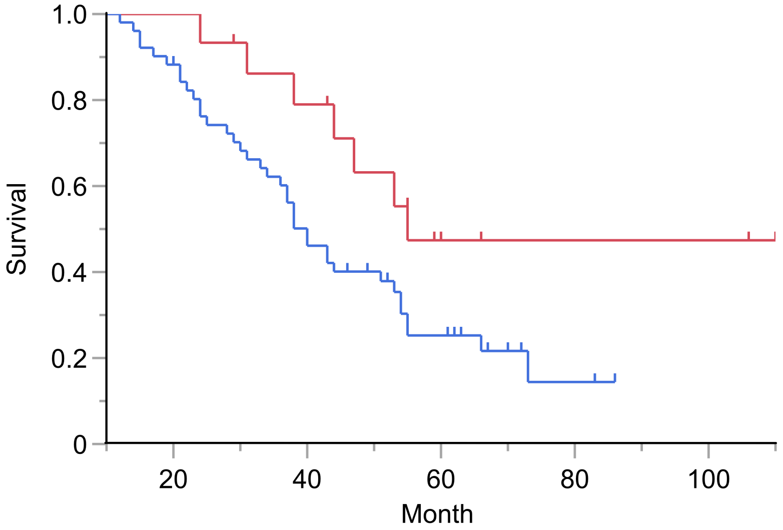


**Supplementary Figure S1.**

Kaplan-Meier plots of survival probabilities according to SDTC. Amyotrophic lateral sclerosis (ALS) patients (n = 66) were divided into higher (red line) and lower (blue line) groups according to the cut-off value of SDTC (0.41 ms). Abbreviations; SDTC = strength duration time constant.

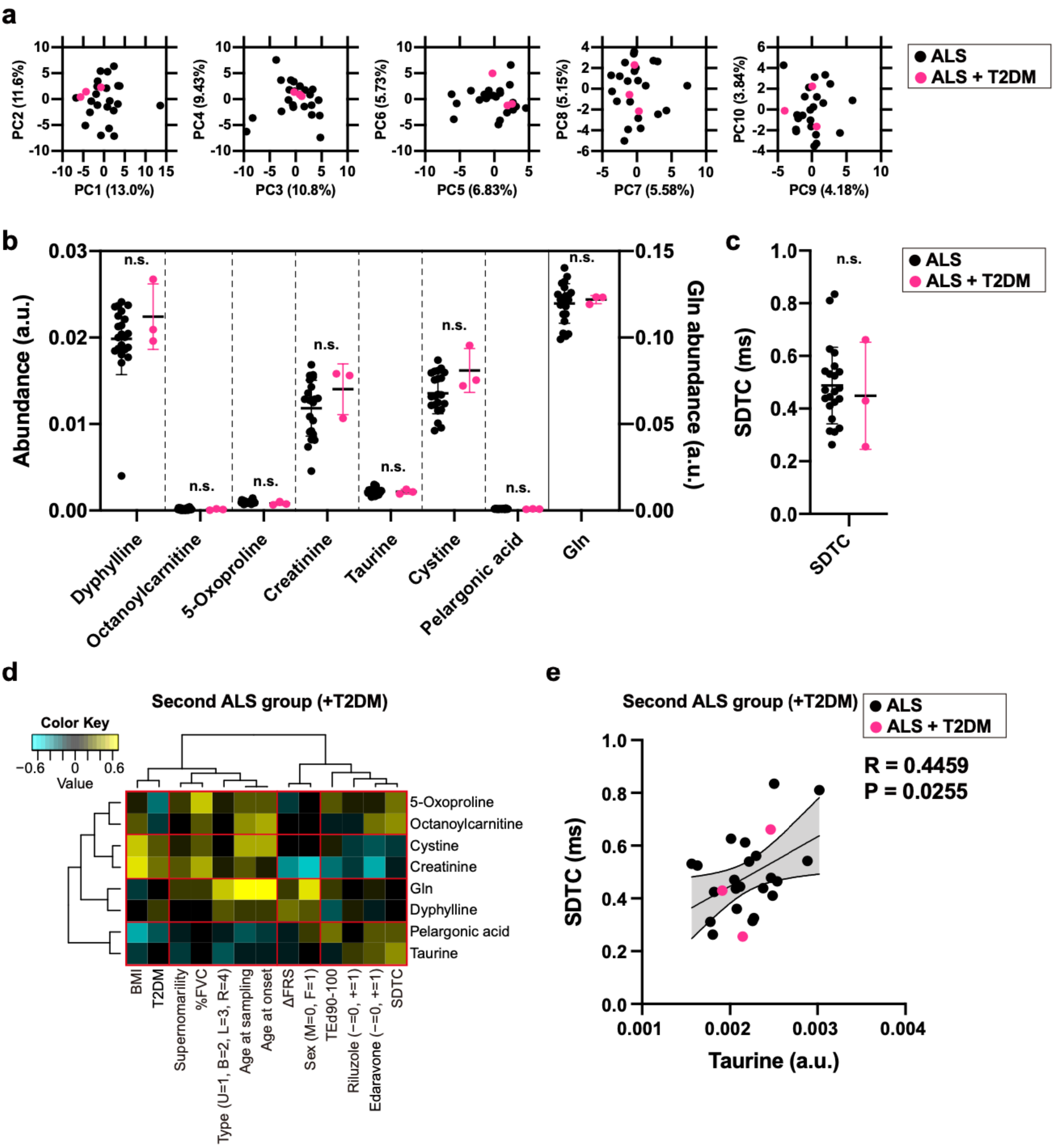


**Supplementary Figure S2.**

(a) Principal component analysis (PCA) of plasma metabolites in the second amyotrophic lateral sclerosis (ALS) group. Two-dimensional plot of PCA scores from PC1 to PC10 in ALS patients with type 2 diabetes mellitus (T2DM) (red, n = 3) versus ALS patients without T2DM (black, n = 22) as analyzed according to the normalized values of plasma metabolites detected in 25 ALS patients consisting of 3 patients with T2DM and the second ALS group (n = 22). (b, c) Abundance of 8 metabolites, a subset of the 17 metabolites with

statistically significant difference between control and the first ALS group (b), and nerve excitability properties (SDTC) (c) detected in the 25 ALS patients. Data are shown as mean ± standard deviation with individual values. P values were calculated by Wilcoxon test. n.s., non-significant. (d) Hierarchical clustering analysis with heatmap representation of the metabolites and the clinical parameters of the 25 ALS patients. Rows indicate a subset of the 8 metabolites described above detected in the 25 ALS patients. Columns indicate the 13 clinical parameters assessed in the study. The heatmap shows a gradient color scale ranging from cyan to black to yellow, indicating the normalized score (Z-score) calculated from Spearman's rank correlation coefficient for each combination. (e) Correlation analysis between taurine and nerve excitability properties (SDTC) in the 25 ALS patients. Scatterplots show the correlation between taurine and SDTC in ALS the 25 ALS patients. Pearson's correlation coefficient (R) and the p-value (*p*) are shown. Abbreviations: a.u. = arbitrary unit; BMI = body mass index; %FVC = percent forced vital capacity; Gln: glutamine; PC = principal component; SDTC = strength duration time constant; Sex (M = male; F = female; TEd = depolarizing threshold electrotonus; treatment with Riluzole and/or Edaravone (- : negative/non-administration; + : positive/administration); type = site of symptom onset (U = upper limbs, B = bulbar, L = lower limbs, R = respiratory failure); ΔFRS = progression rate (ratio of Functional Rating Scale score to time).

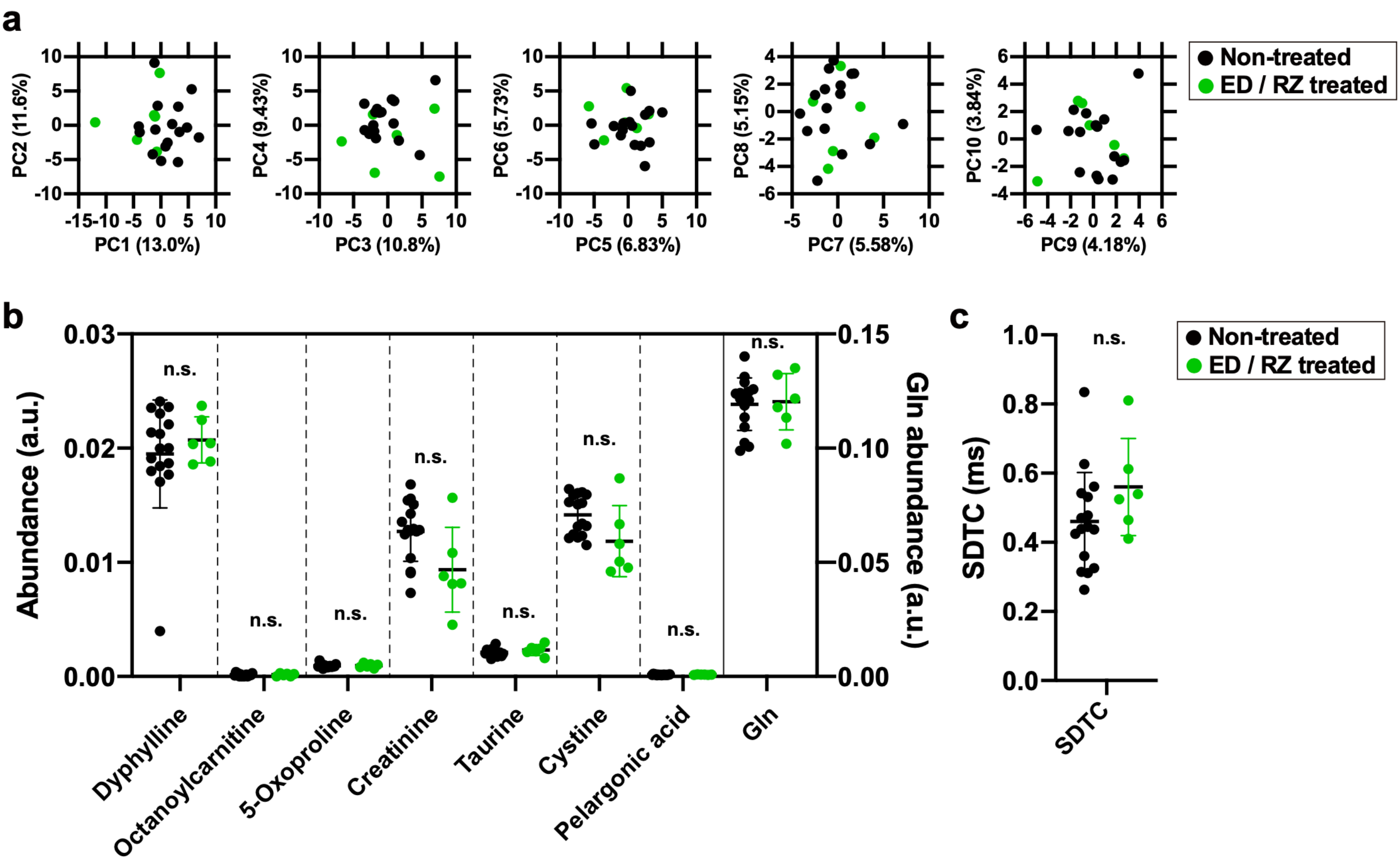


**Supplementary Figure S3.**

(a) Principal component analysis (PCA) of plasma metabolites in the second amyotrophic lateral sclerosis (ALS) group. Two-dimensional plot of PCA scores from PC1 to PC10 in ALS patients with administration of Edaravone (ED) and/or Riluzole (RZ) (green, n = 6) versus ALS patients without ED/RZ treatment (black, n = 16) as analyzed according to the normalized values of plasma metabolites detected in participants in the second ALS group. (b, c) Abundance of the 8 metabolites, a subset of the 17 metabolites with statistically significant between control and the first ALS group (b), and nerve excitability properties (SDTC) (c) detected in the second ALS group. Data are shown as mean ± standard deviation with individual values. P values were calculated by Wilcoxon test. n.s., non-significant. Abbreviations: a.u. = arbitrary unit; Gln: glutamine; PC = principal component; SDTC = strength duration time constant.
